# Outbreak of Trichinellosis Caused by *Trichinella papuae*, Thailand, 2006 

**DOI:** 10.3201/eid1412.080800

**Published:** 2008-12

**Authors:** Chowalit Khumjui, Pravit Choomkasien, Paron Dekumyoy, Teera Kusolsuk, Wandee Kongkaew, Mutita Chalamaat, Jeffrey L. Jones

**Affiliations:** Ministry of Public Health, Bangkok, Thailand (C. Khumjui, P. Choomkasien, W. Kongkaew, M. Chalamaat); Mahidol University, Bangkok (P. Dekumyoy, T. Kusoluk); Centers for Disease Control and Prevention, Atlanta, Georgia, USA (J.L. Jones)

**Keywords:** Trichinellosis, Trichinella papuae, Thailand, foodborne, outbreak, dispatch

## Abstract

In 2006, the Thailand Ministry of Public Health studied 28 patients from a village in northern Thailand. All had myalgia, edema, fever, and gastrointestinal symptoms; most had eaten wild boar. A muscle biopsy specimen from a patient showed nonencapsulated larvae with a cytochrome oxidase I gene sequence of *Trichinella papuae*.

Trichinellosis is a foodborne parasitic zoonosis distributed worldwide that has not always been recognized for its importance, particularly in resource-poor countries (*1*). In northern Thailand, the main source of infection is domestic pigs kept by villagers (*2*). In addition to raising pigs, villagers hunt wild boar and barking deer to supplement their diet. The causative agent of most trichinellosis outbreaks in Thailand has been *Trichinella spiralis* (*3*). However, in 1994, an outbreak of *T*. *pseudospiralis* occurred (*4*).

*T*. *papuae* was described in 1999 (*5*) and has only been detected in Papua New Guinea (*6*). No human outbreaks of trichinellosis caused by *T*. *papuae* have been reported. On July 18, 2006, a cluster of 3 patients hospitalized with myalgia, edema, and eosinophilia was reported from Ban-rai district of Uthai Thani Province in Thailand. Local public health teams found an additional 16 villagers with similar symptoms and launched a full investigation on July 19.

## The Study

The investigation team reviewed medical records, interviewed hospitalized patients, and performed active case finding in the implicated village by visiting the houses of the known patients and inquiring about symptoms of family members and neighbors. A suspected case-patient was defined as a resident of village A who had myalgia and facial, periorbital, trunk, or limb edema during May–June 2006. A confirmed case-patient was defined as a person who met the criteria of a suspected case-patient and who had eosinophilia and a positive serologic test or muscle biopsy result for *Trichinella* spp. A request for volunteers to serve as controls for the investigation was announced by the village president. A control was defined as a resident of village A who did not meet the case definition and who agreed to give an interview, allowed specimen collection, and had negative serologic results for *Trichinella* spp.

Physicians in the Thai Bureau of Epidemiology’s Field Epidemiology Training Program and the Uthai Thani Provincial Health Offices conducted a case–control study to determine the source of infection. A questionnaire was used to collect demographic characteristics, signs and symptoms, date of illness onset, laboratory results, and suspected exposures before onset of illness. An environmental study was conducted to investigate suspected food items, the surrounding area, and wild and domestic animals. Data analysis was performed by using Epi Info 2002 version 2 (Centers for Disease Control and Prevention, Atlanta, GA, USA).

For laboratory investigation, samples were collected from case-patients and controls for complete blood counts and serologic testing. In the hospital, laboratory results, including complete blood counts and creatine phosphokinase (CPK), were reviewed and blood for detection of antibodies to *Trichinella* spp. was collected. A muscle biopsy of 1 case-patient was performed.

Species identification by PCR was performed at the Department of Helminthology, Mahidol University. Briefly, a genomic DNA sample was obtained by using a tissue protocol (QIAamp DNA Mini Kit; QIAGEN, Valencia, CA, USA). The partial cytochrome oxidase subunit I (COI) region was amplified by primers, which were designed from partial COI sequences of mitochondrial DNA from *T*. *zimbabwensis* and *T*. *papuae* in GenBank (accession nos. DQ007900 and DQ007899, respectively). Primers for amplification were Tri-COIF (forward: 5′-GTTTATAT(C/T)(C/T)TAGTACTA CC-3′) and Tri-COIR (reverse: 5′-GC(G/A)TTTGATAGTCT(A/G)ACTCC-3′). DNA alignment analyses were conducted by using ClustalW version 1.83 (www.ebi.ac.uk/clustalw), and nucleotide substitutions were identified by using BioEdit version 7.0.1 (Isis Pharmaceuticals, Inc., Carlsbad, CA, USA) (*7*).

The investigation team also collected blood from domestic boar for antibodies to *Trichinella* spp. and a sample of fermented barking deer meat for larvae examination. No uncooked wild boar meat was available for laboratory analysis. All larvae and human serum specimens were sent to the Department of Helminthology, Mahidol University, Thailand. Immunoblot tests for human trichinellosis were conducted by using the 109-kDa diagnostic band (sensitivity 100%, specificity 100%) (*8*). Boar serum specimens were also tested. Studies were exempted from human subjects review because they were conducted under the authority of the Ministry of Public Health to investigate outbreaks of illness.

**Figure 1:**
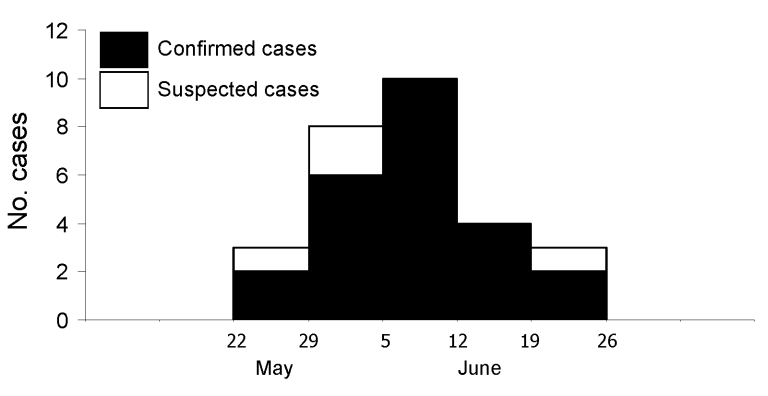
Epidemic curve showing distribution of cases of infection with *Trichinella* spp., by onset date, Village A, Uthai Thani Province, Thailand, May–June 2006.

The study village is located in a mountainous area and had a population of 273. Of the 82 participants, 28 (3 hospitalized patients and 25 other villagers) had an illness that met the case definition (4 were suspected cases). Of the 28 case-patients (median age 34 years, range 14–55 years), 18 were male. The attack rate in the village was 10.3% (28/273); there were no deaths. The outbreak occurred during May 24–June 26, 2006, and the epidemic curve was characteristic of a common point source (Figure 1). All case-patients had myalgia. Other clinical symptoms included trunk/limb edema (83.3%), weakness (75.0%), periorbital/facial edema (70.8%), fever (37.5%), nausea/vomiting (29.2%), jaw pain (20.8%), abdominal pain (12.5%), and diarrhea (8.3%). Mebendazole (plus prednisolone if the symptoms were severe) (*9*) was prescribed for all symptomatic patients.

The environmental study found that villagers were avid hunters of deer, boar, and other local game; they also raised domesticated pigs (the village had 8 domesticated pig sties, each containing 3–10 pigs). Villagers shared domesticated pig meat from various sties every 2–3 weeks. On May 20, a successful wild boar hunt resulted in distribution of wild boar meat to villagers. On May 21, domesticated pig meat was shared among villagers. A successful hunt for barking deer also obtained meat that was prepared in various styles, including cooked, raw, and fermented, and was distributed to villagers during April and May.

**Figure 2:**
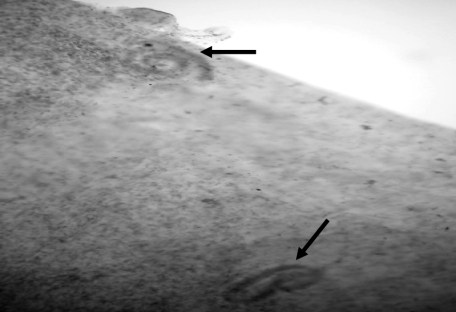
Nonencapsulated *Trichinella* spp. (*Trichinella papuae*) larvae (arrows) in the left gastrocnemius muscle of 1 case-patient, Uthai Thani Province, Thailand, 2006 (magnification ×40).

After eating suspected foods on May 20–22, the first case-patient developed symptoms on May 24 (diarrhea and abdominal pain). Eighty-two blood samples were collected during the investigation; 32 (39%) had antibodies to *Trichinella* spp. Among 28 blood samples from case-patients, all had eosinophilia (>10% eosinophils, mean 28.6%, SD 13.5%) and 21 had leukocytosis, (mean 14,500 cells/mm^3^, SD 4,230 cells/mm^3^). All 3 hospitalized case-patients had elevated CPK levels (median 830 U/L, range 506–1,208 U/L, reference <50 U/L). One domesticated pig was randomly selected from each of 8 pigsties and tested by ELISA for antibodies to *Trichinella* spp.; all pig samples were negative. A human gastrocnemius muscle biopsy specimen from a hospitalized case-patient was positive for nonencapsulated *Trichinella* spp. larvae (Figure 2), which provided a definitive diagnosis of trichinellosis. The parasite had a COI partial gene sequence of *T*. *papuae* (*7*). The case-patient had not traveled outside of Thailand. The fermented barking deer meat was negative for larvae.

Of 28 persons whose illness met the case definition, 24 (3 hospitalized case-patients and 21 villagers) had antibodies to *Trichinella* spp. and were included in the analytic study. Of the 54 villagers who did not meet the descriptive case definition, 46 had negative serologic test results and were included as controls. Eating uncooked wild boar from the hunt showed the highest risk for illness (odds ratio [OR] 9.6, 95% confidence interval [CI] 3.0–30.1) (Table 1). Associations were not significant for eating uncooked domesticated pig (OR 1.8, 95% CI 0.6–5.9) or uncooked barking deer (OR 2.2, 95% CI 0.6–8.4). Subgroup analysis showed that persons who ate uncooked wild boar had a high risk for illness (OR 17.5, 95% CI 3.9–86.0) (Table 2). Although the OR for becoming ill after eating cooked wild boar was increased, it was much lower than that for consuming raw wild boar (OR 2.9, 95% CI 0.4–19.5).

**Table 1:** Univariate analysis of 3 suspected food items in an outbreak of trichinellosis, Uthai Thani Province, Thailand, May 24–June 26, 2006*

Meat ingested	Case-patients (n = 24)			Controls (n = 46)		OR (95% CI)
	Exposed	Not exposed		Exposed	Not exposed	
Wild boar	18	6		11	35	9.6 (3.0–30.1)
Domestic pig	5	19		5	41	1.8 (0.6–5.9)
Barking deer†	19	5		31	15	2.2 (0.6–8.4)

*OR, odds ratio; CI, confidence interval. †Consumed on May 22, 2006.

**Table 2:** Association of wild boar meat and risk for trichinellosis, Uthai Thani Province, Thailand, May–June 2006*

Wild boar meat	Case-patients (n = 24)	Controls (n = 46)	OR (95% CI)
Uncooked	15	5	17.5 (3.9–86.0)
Cooked	3	6	2.9 (0.4–19.5)
Not eaten	6	35	1

*OR, odds ratio; CI, confidence interval.

## Conclusions

Eating undercooked wild boar meat was strongly implicated as the source of this trichinellosis outbreak. The villagers were instructed about the importance of thoroughly cooking meat potentially contaminated with *Trichinella* spp. These findings indicate that the geographic range of *T*. *papuae* is greater than previously thought.
